# Real-time estimation of the effective reproduction number of COVID-19 from behavioral data

**DOI:** 10.1038/s41598-023-46418-z

**Published:** 2023-12-05

**Authors:** Eszter Bokányi, Zsolt Vizi, Júlia Koltai, Gergely Röst, Márton Karsai

**Affiliations:** 1 Institute of Logic, Language and Computation, University of Amsterdam, 1090GE Amsterdam, The Netherlands; 2 National Laboratory for Health Security, University of Szeged, Szeged, 6720 Hungary; 3 National Laboratory for Health Security, Centre for Social Sciences, Budapest, 1097 Hungary; 4 Faculty of Social Sciences, Eötvös Loránd University, Budapest, 1117 Hungary; 5 Department of Network and Data Science, Central European University, 1100 Vienna, Austria; 6 National Laboratory for Health Security, Alfréd Rényi Institute of Mathematics, Budapest, 1053 Hungary

**Keywords:** Complex networks, Computational science

## Abstract

Monitoring the effective reproduction number $R_t$ of a rapidly unfolding pandemic in real-time is key to successful mitigation and prevention strategies. However, existing methods based on case numbers, hospital admissions or fatalities suffer from multiple measurement biases and temporal lags due to high test positivity rates or delays in symptom development or administrative reporting. Alternative methods such as web search and social media tracking are less directly indicating epidemic prevalence over time. We instead record age-stratified anonymous contact matrices at a daily resolution using a longitudinal online-offline survey in Hungary during the first two waves of the COVID-19 pandemic. This approach is innovative, cheap, and provides information in near real-time for estimating $R_t$ at a daily resolution. Moreover, it allows to complement traditional surveillance systems by signaling periods when official monitoring infrastructures are unreliable due to observational biases.

## Introduction

Behavioral patterns strongly influence the outcome of an epidemic, yet observing how they change during an unfolding pandemic is among the largest challenges^1, 2^. Alongside conventional survey methods, recent online and digital technologies provide new solutions to this problem. However, it is not evident how to translate large-scale observational data into actionable input for operational processes such as epidemic surveillance or modeling. Moreover, the dynamical estimation of social interaction patterns for large representative populations is problematic without entering privacy issues. We built an online/offline data collection infrastructure to continuously follow age-stratified contact matrices in a large population during the COVID-19 pandemic^3^. Integrating these self-reported contact numbers from voluntarily provided anonymous online questionnaires into disease transmission models, we demonstrate how to estimate the dynamics of the effective reproduction number from behavioral data. Alongside the conventional solutions based on medical statistics and population testing, our ecosystem provides a complementary surveillance system for disease monitoring.

Behavioural responses to pandemic emergencies. There are several reasons why people change the way they interact, travel, or protect themselves during a pandemic. Non-pharmaceutical interventions (NPIs)^4^ such as lockdowns, school closures, mask mandates, and other regulations are the most direct causes that might induce change in people’s behavior. However, fear of contamination^5^, lack of trust in governmental communication^6^, or belief in misinformation^7^ can also cause a radical shift in one’s social and mobility patterns, sometimes even leading to counter-effective situations like mass protests against regulations in the middle of a pandemic^8^. Therefore, it is challenging to dynamically observe the convoluted effects of all these behavioral forces, not to mention their explanation by disentangled causal reasons.

It is essential to understand how people alter their social behavior^3, 9, 10^ and mobility patterns^11–13^ during a pandemic^2, 3, 14–17^. These changes directly influence the way people meet, mix and interact with others, which then determines the dynamics of the disease spreading. The follow-up of direct physical contacts or proximity interactions of people are crucial from an epidemiological point of view as they provide the underlying conditions to transmit various types e.g. influenza-like illnesss^9, 18–20^. Therefore, the social networks of people that encode physical and proxy interactions might provide critical input to epidemic models at different levels of aggregation, like in forms of age-stratified contact matrices^9, 21–23^. Even though the dynamical monitoring of such social networks is a prime goal during epidemic crises, conventional methods like phone recorded surveys and contact diaries may have limited capacities to capture relevant information in a reflexive way. This is especially the case in the beginning of a pandemic emergency when immediate data collection is crucial, while phone surveys could provide observations through expensive and highly coordinated efforts only^24–26^. Nevertheless, one such achievement has been carried out during the CoMiX study^27^ that aimed to recurrently collect age stratified contact patterns in several European countries during the COVID-19 pandemic. This panel survey study recorded data about the contact patterns of people influenced by local interventions during the pandemic by registering age contact matrices in different contexts^28^. Such dataset fuelled studies like Munday et al.^29^, that aimed to develop predictive epidemic models incorporating the dynamically changing contact matrices in the UK, and established the observations that they improve the forecast of infections in the short and mid-term time-horizon during winter months, especially for children and older adults. However, beyond these conventional data gathering techniques, innovative solutions exploiting novel digital data collection methods can be designed to follow people’s social dynamics. Such methods may rely on online social platforms, contact tracing apps, or online questionnaires^23, 30^, as it will be demonstrated in this paper.

### Epidemic surveillance methods and their biases

One of the broadly adopted metrics to characterize the actual state of an epidemic is the *basic reproduction number*^31^ ${R_0}$. This measure determines the expected number of secondary infection cases induced by a single infected individual in a fully susceptible population. If this number $R_0>1$, the number of confirmed cases will rise, whereas if $R_0<1$, there will be no sizeable outbreak. Nevertheless, during an evolving real epidemic with a large fraction of infected people, the spreading dynamics is better estimated by the *effective reproduction number* $R_t$. This quantity takes the actual size of the remaining uninfected population into account and incorporates all other aspects that influence the course of the epidemic. It is affected by several factors, such as the transmission rate of the infection, the duration of infectiousness of infected individuals, or the contact frequency in the host population^32^.

For a given population, $R_t$ is usually calculated with statistical tools^33, 34^ from epidemiological data like the number of fatalities or the detected number of infected cases. These numbers are collected via centralized national surveillance systems, which are expensive to maintain and may rely on data reporting practices that are not always transparent. Moreover, none of these observables provide a good solution to nowcast the actual $R_t$ values. Fatalities are usually well documented, thus, their count could potentially provide a precise measure to estimate $R_t$. However, identified COVID-19 deceased are reported usually with delays after their initial infection, due to the different course of the illness for different individuals, and also due to reporting delays. Such delays fluctuate and can mount up to weeks, which makes fatality counts impossible to use for the real-time monitoring of the epidemic. The number of detected cases are usually reported more rapidly but they provide less precise observables. These counts easily fluctuate due to extreme events or other biases. One of their most significant observational bias is caused by limited testing capacities, inducing high *positivity rates*. Following the recommendation of the World Health Organization (WHO), the test positivity rate should not exceed $5\%$^35^ for reliable observations. However, during the early phase of the pandemic, due to the shortage of tests and later upon the emergence of highly transmissible variants, this condition was difficult to maintain. This caused severe underestimation of $R_t$ during major epidemic waves in many countries^36^. Other biasing factors come from case importations and local epidemic clusters, testing campaigns, or the slow data retrieval due to delayed case reporting. All these shortcomings make these conventional observables difficult to use for the precise and real-time inference of the actual $R_t$ values during an emerging pandemic. This calls for novel methods to estimate $R_t$ dynamically from alternative data sources in order to provide independent monitoring tools to follow the actual epidemic and to help operative decisions.

### Behaviour dynamics for epidemic survelliance

To answer this challenge, we have built an infrastructure that can estimate the effective reproduction number $R_t$ in real-time with remarkable precision using contact dynamics data collected online and via telephone surveys. More precisely, we collected daily age-stratified contact matrices during the first and second waves of the COVID-19 pandemic in Hungary using an online questionnaire, which was answered 538, 684 times by 235, 072 unique users since its launch. Meanwhile, we recorded the same questionnaire each month on a representative population of 1, 500 individuals via telephone surveys (for more details, see Methods). With the combination of the two datasets, we reconstructed a sequence of age contact matrices at a daily resolution, that we share through an open repository^37^ along this paper. In turn we feed these matrices as an input to a deterministic epidemic compartment model, which this way not only considers the age-stratified contact patterns of the modeled population but incorporates the effects of contact behavioral changes in its dynamics. The numerical solution of this model served us with an inferred $R_t$ function at a daily resolution, that closely approximated the $R_t$ values computed from hospitalization rates for a reference period.

Despite all these advantages, the behavioral monitoring based $R_t$ inference method appears with biases too, and may provide obscure data in certain cases. For example, if the number of recorded responses become very low during a period of data collection, or the respondent population will become non-representative for the country, the inferred contact numbers and the estimated $R_t$ value will also become biased. We propose statistical methods to account for these imperfectnesses, yet they may not be able to account for all of them. Examples are social desirability and selection biases, which may be present during the data collection and we assume that they do not change over time. However, this might not be the case, especially over longer periods of recording. As a consequence, instead of suggesting our system as a standalone methodology, the surveillance and behaviour based monitoring systems estimating the dynamics of the $R_t$ reproductive numbers should be observed together as they are independent thus they could indicate observational biases either way if they deviate from each other.

Consequently, our solution provides a cheap supplementary monitoring system complementing observations made via conventional surveillance infrastructures relying on the public health system. It allows for cross-validating and indicating weaknesses of official surveillance when the inference of $R_t$ is biased. Moreover, our monitoring method can closely follow the effects of NPIs on contact numbers of individuals, thus allowing us to evaluate the impact of regulations in almost real-time. In the Results section, first, we briefly describe our data collection, integration, and modeling infrastructure. Subsequently, we present our findings on the observed contact dynamics in Hungary and the reconstructed $R_t$ function that we compare to the official surveillance data. Finally, we discuss the potentials, limitations, and future directions of our results.

## Results

### Data collection and pre-processing

Ten days after the first officially reported COVID-19 case in Hungary, an online data collection platform was initiated to track the social and individual behavioral changes of people during the unfolding pandemic^3^. The so-called Hungarian Data Provider Questionnaire (“Magyar Adatszolgáltató Kérdöív” - MASZK)^38^ data collection started on March 23, 2020 and has been continued ever since. Over this period, the online questionnaire has been answered 538, 684 times by 235, 072 unique users, which is roughly $2.4\%$ of the total population of Hungary.

**Figure 1:**
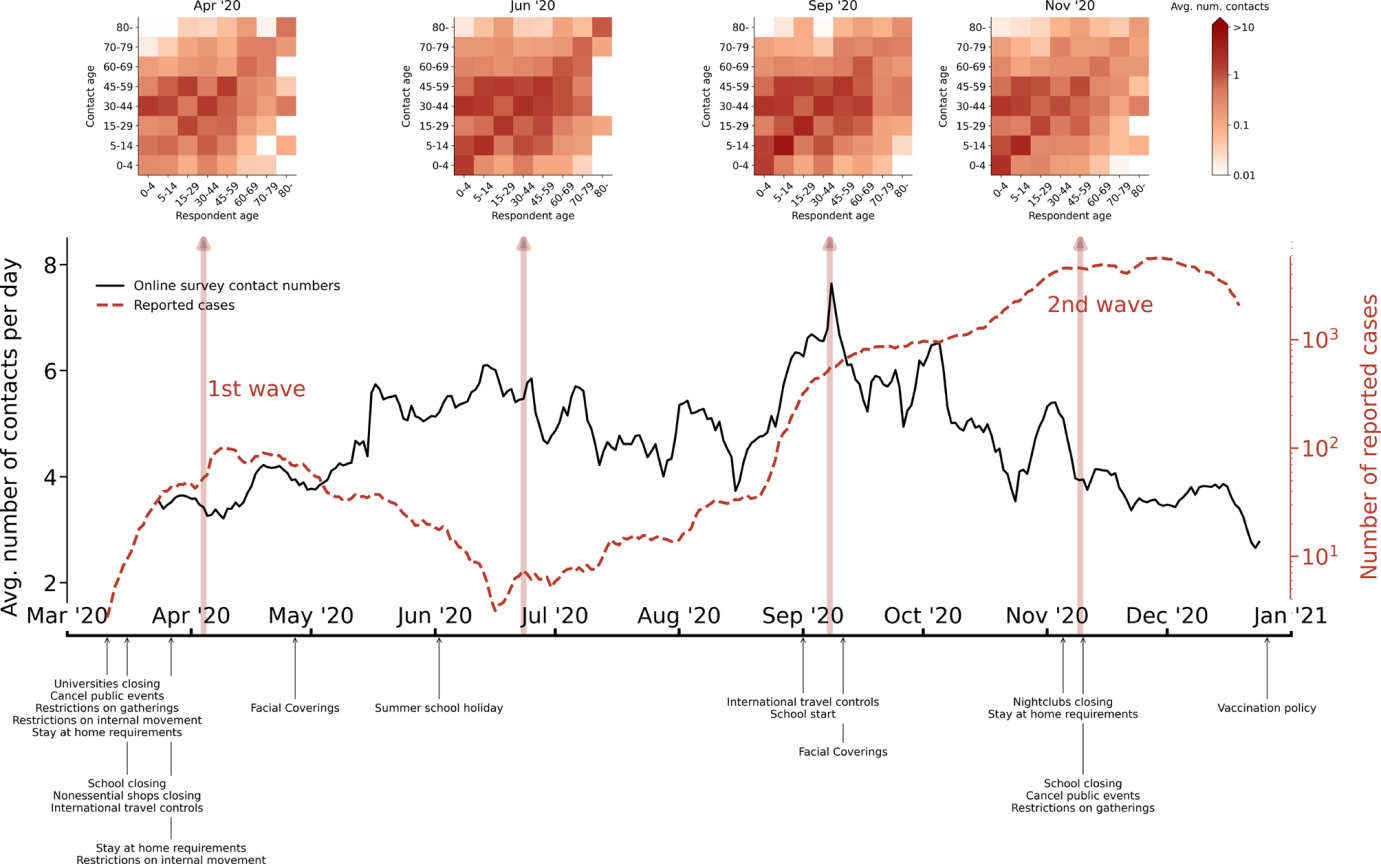
Average contact numbers calculated from the online survey (black line), parallel to the number of confirmed cases in the same period (red dashed line). The timeline of the most important NPI measures in Hungary is below the horizontal axis^39^. Case numbers are smoothed by a 7-day sliding window, similarly to the calculated average contact numbers, that also aggregate online survey data into 7-day sliding windows. Four selected contact matrices for the 8 age groups are shown above the curves. The effects of lockdowns and school closures (or the lack of them) are evidently visible in the matrix elements.

Respondents were asked to estimate the number of people from eight different age groups ($0-4$, $5-14$, $15-29$, $30-44$, $45-59$, $60-69$, $70-79$, and $80+$) they got in contact with during the previous day without mask protection. Such *proxy* contacts were defined as having spent more than 15 minutes within 2 m distance with someone, while at least one of them being without a mask. Relevant to this study, people also provided several of their socio-demographic characteristics (e.g. their age, gender, education level, resident municipality, etc.). Although the data collection involved only adult participants (over the age of 18), parents were asked to give their estimations about the contact numbers of their underage family members. As reference period, responses were also recorded about respondents’ contact patterns from the period before the COVID-19 pandemic. In the actual study, we limit our observations to the first two epidemic waves in Hungary, falling between the 1st April and 31st December 2020, during which the same virus variant was dominantly spreading and vaccination was still not available.

Although a large number of people participated in the data collection, since the online questionnaire was fully voluntary and anonymous, it did not provide a representative sample of the whole population of the country. We addressed this problem by collecting the same questionnaire in parallel using a phone-assisted survey method on a monthly basis. The interviewed population of this survey was representative for the Hungarian population along several dimensions, namely age, gender, settlement type, and education level. We summarize this data collection pipeline in the Methods section in Fig. 3 with more details on data collection, filtering, and pre-processing^23^. As a result, using the daily questionnaires asking about the respondent’s contact patterns during the preceeding day, we could reconstruct daily contact matrices (using a statistical method explained in Methods) and follow the average number of contacts per person over the course of the pandemic, as demonstrated in Fig. 1 for the first two epidemic waves. Asking respondents to recall their contact numbers from the preceeding day minimised the recall bias of the estimates and provided us contact numbers with small memory errors. To mention as an interesting reference point, for the pre-pandemic period, we measured roughly 19.2 contacts per a person on an average day, that was estimated from answers recorded between 1 April 2020 and 1 June 2020, asking about contact numbers before 23 March 2020, averaged similarly as explained in Methods section explaining contact matrix reconstruction. This number drastically reduced by more than $80\%$ in 2020 March, after which contact numbers conversely followed the actual number of infected cases in the country, as shown in Fig. 1. Note that while we share this pre-pandemic contact number as an interesting reference point, this number may suffer from stronger recall biases, thus it is never used in our analysis and modelling.

### Estimation of the effective reproduction number

We used the obtained daily contact matrices as an input for modeling the transmission dynamics. We estimated the time-varying reproduction number during the course of the epidemic waves by employing a deterministic compartmental model. This model contains classes for latency, infectious and hospitalized period, and relaxes the condition for homogeneous mixing via tracking transmission routes between age groups in the population. For the visual representation of all transitions between the compartments, see SI Figure 2 and for the system of resulted equations, see SI equation (1). To incorporate age-stratified transmission patterns, we used the previously computed dynamical daily contact matrices. They represent the heterogeneity of the social contacts among individuals of different age groups, thus, they form the basis for the calculation of the associated effective reproduction number. Further, we considered seasonality effects deeming periodically lower transmission rates of the epidemic during the summer periods. The model includes an age-dependent parameter for susceptibility, which is smaller for young individuals implying less effective transmission. For details about the compartment structure, parametrization, and seasonality integration of the epidemic model, see Supplementary Information.

We iteratively solved the system defined by the model, starting from the state of the previous day and replacing the contact matrix of the next day in the simulation. During the model solution, the time-dependent age vectors of susceptible individuals were used to calculate the effective reproduction number on a daily basis. Since the number of infections during the first wave in Hungary was very low, the significance of tracking the depletion of susceptibles in age groups appeared only in the second wave. Consequently, we started our modeled epidemic from a fully susceptible population in April 2020 and simulated it for nine months, until January 2021, which corresponded roughly to the end of the second pandemic wave in Hungary. We chose a reference period, when the case number based $R_t$ estimate is deemed accurate, and used this reference point to calibrate the relation between social contacts, fraction of susceptibles, and $R_t$ value. The selected reference period was mid-September, since for this period we obtained the most reliable $R_t$ estimate, which was confirmed by different types of epidemiological data, such as incidence data, hospitalizations and mortality trends (see^40^). Having this reference point fixed, following our methodology, we could calculate the $R_t$ rates for periods prior and posterior to the reference point. As a model output, we computed the $R_t$ effective reproduction number using the so-called Next Generation Matrix (NGM)^41, 42^ method, which partitions the model structure to transition (focusing on the flow between the classes) and transmission (involving age-specific social patterns) parts. At a time point *t*, we compute a matrix whose dominant eigenvalue provides the value of $R_t$. For complete description of the methodology, see the Supplementary Information.

Note, that in a GitHub repository we share the epidemic simulation code incorporating the dynamical contact matrices^37^.

**Figure 2:**
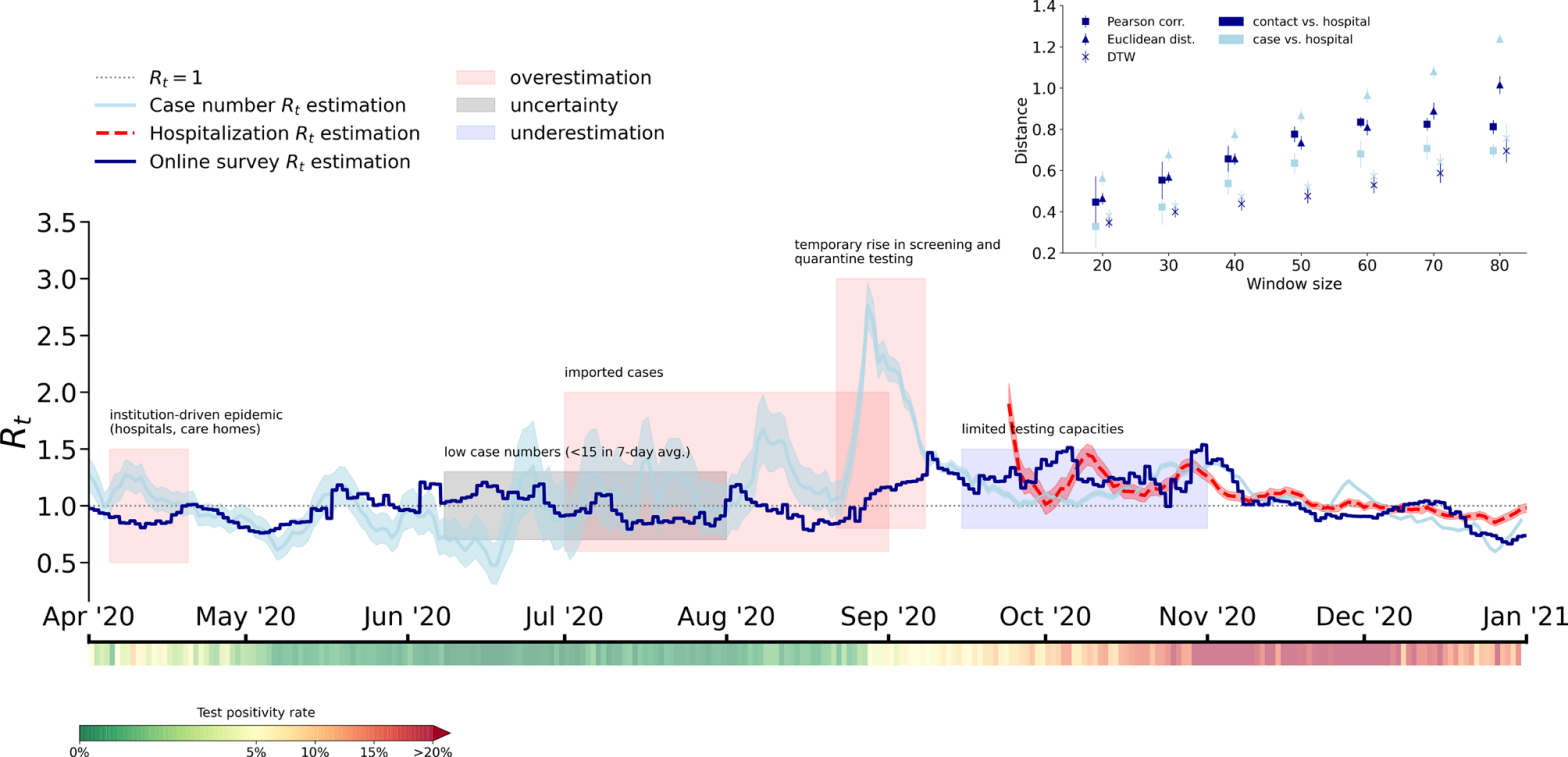
Effective reproduction numbers between 1st April 2020 and 31st December 2020 in Hungary estimated from the daily contact matrices of the online survey (dark blue), and from the case numbers using the Cori method (light blue)^33^ with statistical credible intervals shown as blue shaded area. Reference $R_t$ estimated from the 3rd October 2020 using hospitalization numbers is shown by a red dashed line together with $95\%$ credible intervals. The black dotted line indicates the $R_t=1$ critical reproduction number. Colored stripe below the horizontal axis depicts the test positivity rate as a percentage of positive tests of all tests taken in the country on the given day. Annotated boxes show periods where methods based on case numbers either overestimate (red) or underestimate (blue) the reproduction number, and where the method exhibits uncertainty due to very low case numbers (yellow). The inset presents the comparison of the contact matrix based and case number based $R_t$ estimations to the reference curves based on hospitalization numbers to estimate $R_t$. Differences between curves were measured by the Pearson correlation as a similarity, and Dynamic Time Warping and Euclidean distance as distance metrics with $95\%$ confidence intervals shown.

### Alternative reproduction number surveillance for Hungary

The estimated effective reproduction numbers are shown in Fig. 2 during the first two pandemics waves in Hungary. There, the dark blue curve corresponds to the $R_t$ estimated from our model solutions, which relies on online data and it takes into account the dynamical change of contact patterns. On the other hand, during the same period, several other methods have been proposed and applied to track the effective reproduction number in real time^33, 34^. These estimations commonly rely on the reported case numbers, which suffer from numerous biases, which could even change during the pandemic. We use one such estimate publicly available at^43^ that we indicate by a light blue curve and the corresponding $95\%$ credible interval in Fig. 2. This curve represents an estimation of $R_t$ computed by the Cori method^33^ using the official Hungarian case numbers. While recently other, more up-to-date methods^44–46^ became availaible to estimate $R_t$ from case numbers, we use the actual curve^43^ as this was the only $R_t$ estimation published in Hungary during the pandemic^42^, and served as primary source for situation assessment.

By looking at Fig. 2, both the official and simulated $R_t$ values were smaller than one in the spring of 2020, confirming that the first wave was successfully suppressed. It was hovering around one during the summer and started grow distinctly above one from September onward, signifying a large second wave. The $R_t$ value dropped below one at the end of November, marking the peak of the second wave, and remained below one afterwards, indicating the decay phase of the second wave. Generally, the effective reproduction numbers estimated from online data and model simulations were following surprisingly well the officially reported $R_t$ numbers for the entire period. Moreover, given a past reference point, this method allows us to make $R_t$ estimates not only retrospectively, but also in real time, presuming that social mixing data is collected continuously in real time as well.

At the same time, it is evident that the two estimated $R_t$ curves deviate from each other during some periods. We indicate these periods with colored boxes, during which the official reproduction number was deemed less reliable and deviated from the modeled curves. Following a chronological order, the first wave in Hungary in the spring of 2020 was dominated by outbreaks in healthcare and social care institutions. Therefore, these outbreaks generated a sharp increase in reported cases, leading to some short living spuriously high $R_t$ values in the case number based estimate. Yet, although these cases increased the number of confirmed cases, these high values did not represent the spread of the infection in the general population, as they correspond to well-contained local outbreaks^42^. This was captured by the modeled $R_t$ values, which remained under one during this period.

Subsequently, in mid summer 2020, reported values have been noisy due to low case numbers (<10, yellow period in Fig. 2). This explains the very low numbers of case based $R_t$ numbers, as compared to the modeled values, which remained higher due to the relatively large number of social contacts during the summer. After lifting the border closure measures, during the late summer, there was a period of time when the infection numbers were driven by case importations from abroad, inflating again the $R_t$ estimate above the modeled values. From mid august 2020, the government carried out a large screening campaign in freshmen camps before the start of the higher education autumn semester. This has lead to an artificial peak in the infected case number based curve. Meanwhile, the convoluted effects of case importations, mass events like weddings and freshmen camps, the increased social contact numbers due to the beginning of the school year, and the seasonally augmented transmission rates led to the emergence of the second wave in Hungary. This was actually well reflected by the modeled curve using contact numbers that signaled increasing $R_t$ numbers from 2020 September.

In the exponential phase of the second wave, Hungary quickly reached its limit in testing capacity, and the reported case numbers did not grow any further. This resulted in a misleadingly low $R_t$ estimate in the case number based curve^40^ in October 2020. This phenomenon is especially striking in the period when the test positivity rates, indicated by the colored stripe below the horizontal axis of Fig. 2, grew steadily from the beginning of September until November (blue period on the main panel). Based on the estimation of $R_t$ derived from case numbers, public health authorities did not assess the pandemic situation correctly in this period, which delayed the introduction of more serious NPI measures to control the fast spreading. Interestingly, from our alternative surveillance, we observed more realistic $R_t$ numbers that were significantly higher than one during this period. The case number based and online estimated $R_t$ curves matched again once the test positivity rate reached a stationary value around the stagnation period of the second pandemic wave from November 2020. Consequently, if we compare our contact number based $R_t$ estimations to the case number based approach, we can see that in the indicated periods that suffer from one of the aforementioned problems, we have a better estimate than the reference curve.

### Validation of inferred reproduction numbers

Apart from the previously cited limitations, the case number based $R_t$ estimations also suffer from the time lag that comes from the disease course (most cases turn positive once the patients have symptoms), and the delays in sample processing and test reporting procedures. The identification and correction of the biases in these case number based estimations require tremendous epidemiological work, high quality data on each individual case beyond raw case numbers, and an intimate knowledge of the country’s surveillance and reporting system. On the other hand, hospitalization numbers, Intensive Care Unit (ICU) admission rates, or the number of deaths have even larger time lags due to the temporal disease progression. Nevertheless, these numbers are more reliable and indicative of the spreading than the reported number of confirmed cases.

We use such an $R_t$ curve, estimated from daily hospitalization counts, to validate whether the contact number based or the case number based $R_t$ curves meet closer with the reference. The hospitalization number based $R_t$ curve (red dashed line in Fig. 2) was collected only after 2020 October, as data from earlier periods are not available, but it has been argued to be insensitive to testing biases^40^. We performed pairwise comparisons between the case number vs. hospital number based and the online contact numbers based vs hospital number based curves. To compare these temporal sequences we used multiple metrics: the Pearson Correlation as a similarity measure, and the Euclidean Distance and the Dynamical Time Warping as distance measures. Note that with these measures we were not aiming to infer any causal relationships between these signals, but only quantifying their similarities. Comparisons were made by using sliding time windows with different sizes, indicated as the x-axis scale in Fig. 2 inset. There we see that the contact number based $R_t$ curve is significantly more similar for any window size to the hospitalization based reference curve as compared to the similarity of the case number based estimates.

Although we could demonstrate that the contact number based $R_t$ estimates approximate the reference values better, our goal with these proposed methodology was not to replace official surveillance results using case numbers for their estimates. We rather aimed to propose alternative surveillance observations that complement the official monitoring tools. Remarkably, in the 2020 autumn period of growing test positivity rates, our estimation remains above one, indicating a fast growing epidemic. This highlights an important aspect of our methodology provided by monitoring social mixing dynamics, as it allows to overcome some of the biases in the case number based $R_t$ estimations. Interestingly, we can provide an $R_t$ value estimate, which during biased intervals give a better picture about the unfolding epidemics, this way complementing the traditional surveillance system.

## Discussion

Beyond official surveillance relying on detected case numbers and medical statistics, alternative methods can monitor the unfolding of a pandemic^47, 48^. Some methodologies rely on geo-localized web search and social media tracking to nowcast trends in epidemic-related topics^49, 50^. Online voluntary data collections are frequently used as a complement to population based surveillance^51–53^. Such data is used for the discovery of low detection rate by the surveillance system^54^, proven to be useful in the up-to-date monitoring of virus-based illness^55^, or combined with a realistic data-driven epidemiological model. These data can even be a base for providing good-quality forecasts of epidemic intensities^56^. Despite the popularity of these methods, their vulnerabilities and limitations got evident over the years^57^. In several other studies, the reproduction number of an epidemic is estimated from the mobility patterns of people. Human mobility followed by mobile phone activities, GPS devices, or check-in data could signal the traveling, commuting, and mixing patterns of people, which largely determine the spread of an epidemic in a larger population. However, despite the many promising results^58–60^, the mobility activity of people, quantified by various indices^61–63^, does not always follow the epidemic curve of the pandemic. People accept and follow some interventions better, while some others less. Whereas mask use became a worldwide accepted norm, mobility restrictions became less and less enforced and followed. Therefore, the trends of people’s mobility and the number of infections may diverge^64, 65^. Also, statistics, such as the age-stratified mixing patterns or the fraction of recovered population, are hard to follow with mobility data, which prevents the precise estimation of the effective reproduction number using this type of data sources. For all these reasons, although mobility monitoring plays an essential role in estimating mixing patterns, it may appear as a less correlated direct indicator of epidemic prevalence over time. Our modeling approach could provide a more reliable solution, as it integrates dynamical contact information into epidemic models in the form of time-varying age-stratified contact matrices. This way, it directly introduces the effects of interventions and behavioral changes through the recorded dynamics of social interactions, which leads to better approximations of possible transmission events of disease spreading.

Nevertheless, our proposed methodology has certain limitations. Most importantly, it heavily relies on the respondent population size and its representative composition (for the change of representativeness of our data see Supplementary Information). Although by using combined online/offline data collection methods, we accounted for the non-representativeness of the recorded data, this remains a challenge. We found the representative weighting dimensions robust over the observation period, but they may change over time. Thus, repeated data collection campaigns via representative telephone surveys are necessary. At the same time, while we account for seasonality, other environmental factors (like humidity or pollution level) may influence the epidemic outcome. On the other hand, due to the continuous evolution of new genetic variants, although the biological profile of the pathogen (e.g. its transmission rate or the length of the incubation period) may change, that can be considered in our model. We could also incorporate the dynamics of vaccination and waning immunity into our modeling framework. Finally, voluntary responses may suffer from multiple biases, like recall bias, social desirability bias or selection bias. We aimed to decrease recall bias due to memory errors by asking the respondents about a timeframe close to the interview (the previous day). However, this bias is inevitably present, especially among those with high number of contacts who might be not aware the precise age category of all of their contacts. Additionally, social desirability and selection bias can potentially induce over-representative numbers of answers from overly alert people during some time of the pandemic. Such biases are difficult to capture with our demographic variables in the representativity correction process, but as it is described in the Methods section, as we can assume that these bias are consistently present over time during the observation period, we believe that they do not significantly affect the estimations. This assumption might not be precise for the case of social diserability bias as during periods of high infection rates people may report less number of contacts to comply with the official communication emphasising to reduce the number of social contacts. However, during periods, when the infection rate is lower, there might be less social pressure to limit, and thus, to report a lower number of contacts. While our data does not allow to follow the course of this kind of bias, other studies report weak variability of social diserability bias in similar survey studies recorded during the COVID-19 pandemic^66,67^, thus supporting our original assumption approximating this bias as time invariant. At the same time, we have no reason to believe that the degree of bias changed over time in regards to the other aforementioned biases.

The dynamically varying number of social contacts are one of the primary indicators of social mixing that can potentially estimate the transmission rate of an influenza-like illness. To demonstrate this approach, we described a data collection effort to record age-stratified contact matrices in Hungary at a daily resolution. We integrated them into a deterministic compartment model to estimate the temporal evolution of the reproduction number of the COVID-19 epidemic. This innovative solution provides a cheap and near real-time surveillance system independent of public health data. Instead of using contact tracing, frequent representative surveys, or medical statistics, it relies on the combination of alternative data sources collected online and offline with the involvement of thousands of individuals. It provides a powerful solution alongside conventional surveillance systems to cross-validate their results.

The overall goal of NPIs is to suppress the possible epidemic transmission by decreasing the number of contacts of people through different ways of regulations. Our framework provides a way that can directly monitor the effectiveness of these restrictive measures. It allows to immediately evaluate their impact on larger populations compared to behavioral patterns before and after the regulated period. This method provides an inventive tool for disease monitoring with easy implementation in many countries. Beyond its scientific merit, it may provide effective monitoring of the consequences of national interventions, to follow the effects of population-level behavioral changes, and to inform intervention planning and policy design. Moreover, as we demonstrated in the case of Hungary, it allows to complement traditional surveillance systems in two ways: by signaling periods when official monitoring infrastructures are unreliable due to observational biases; and by providing more accurate signals of the epidemic dynamics during these periods. For all these reasons this methodology should be integrated into future public health surveillance systems for more precise epidemic monitoring.

## Methods

### Data collection and reconstruction pipeline

#### Online data collections

We collected data via an online questionnaire^68^ that users could fill using web browsers or mobile phone apps. Our data collection was completely anonymous using local encrypted browser cookies to improve user experience, without requiring participants to share any personal identifier that could be used for their identification. The data collection was fully complying with the actual European and Hungarian privacy data regulations and was approved by the Hungarian National Authority for Data Protection and Freedom of Information^69^, and also by the Health Science Council Scientific and Research Ethics Committee (resolution number IV/3073- 1 /2021/EKU). During our analysis all methods were performed in accordance with these relevant guidelines and regulations.

The responses contained information on the demographics and family structure of the anonymous users, their contact numbers from the previous day by the age of the contacted people in different situations (e.g. indoors, outdoors, at the workplace etc.), and other questions relevant to their behavior during the epidemic (see^23^ for further description of the questionnaire). To define what counts as a contact, as explained in the questionnaire, we considered two people to be connected if they spent at least 15 minutes without mask protection at a distance less than 2 m (proxy contacts) from each other. Household members were automatically counted as contacts (family contacts) using the family members’ age to consider them in the age-contact matrix. Although data was not directly collected about children, adults (typically parents) could fill a special part of the questionnaire to give their estimations about the proxy contact numbers of their underage family members (typically children).

**Figure 3:**
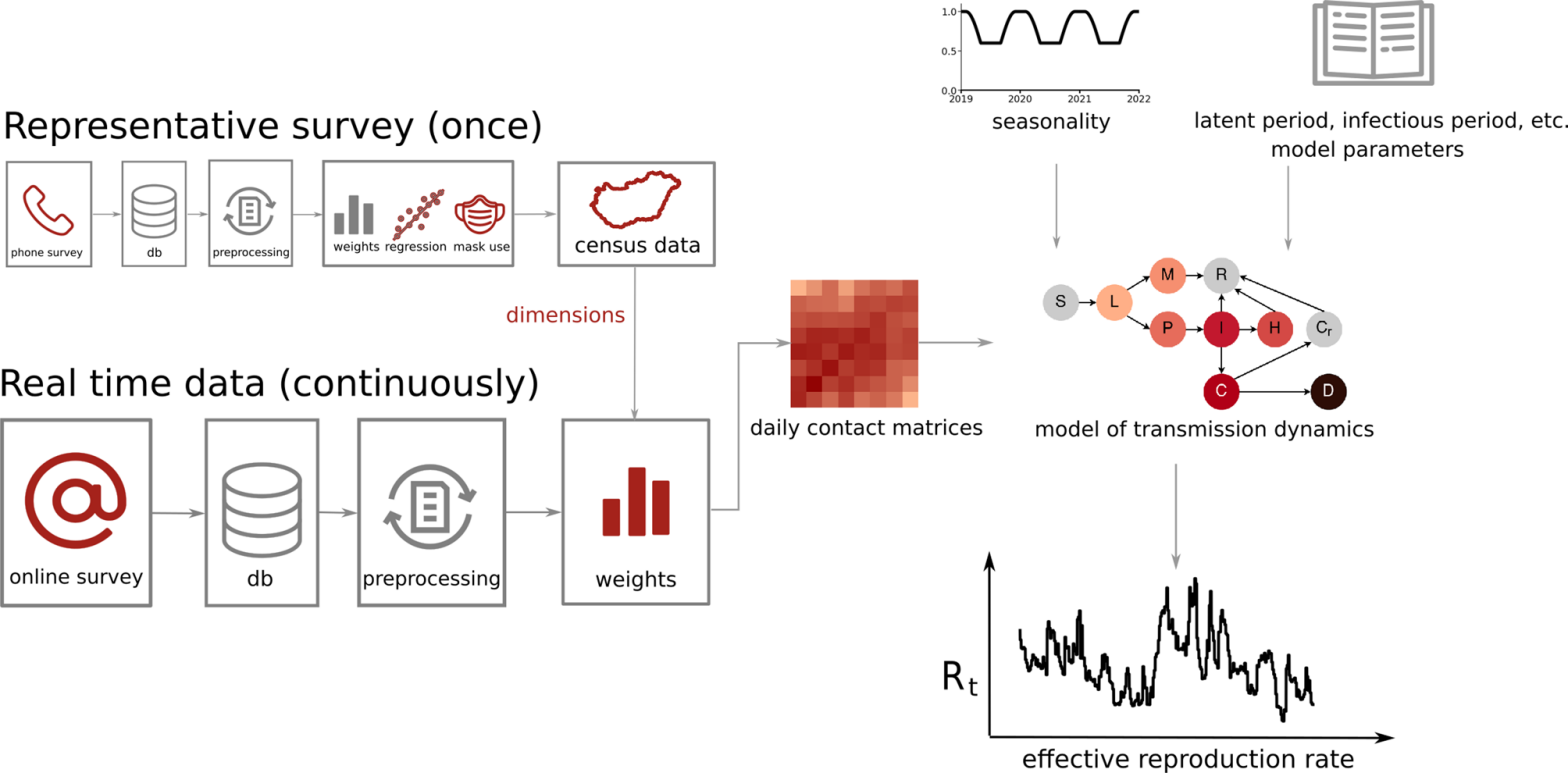
Schematic diagram of the data collection, data processing, and modeling pipelines. The representative phone survey is used for calculating the most important demographic dimensions that influence the average contact numbers of people, and for estimating mask use percentage in different age groups of children. Daily contact matrices are created using a 7-day sliding window from the online survey, adding user weights to correct for sample representativity using the relevant demographic dimensions and their population distribution from official census statistics^70, 71^. Daily contact matrices are then used as input parameters to the compartmental model that uses also biological and medical parameters, as well as a seasonality correction function for the estimation of the daily effective reproduction number $R_t$.

Our observations were focusing on the period from 26/03/2020 to 31/12/2020, during which we collected 429, 267 responses from 230, 878 unique users. To avoid high noise rates, we aggregated daily online answers with a 7-day sliding window that we shifted by one day through the observation period. To make the answering more comfortable for the respondents (and thus, to increase the willingness for participation), we provided intervals for their estimated number of contacts, that we converted to their midpoints before calculations (category conversions were $0:0, 1-2:1, 3-6:4, 7-15:11, 16-30:23,31-60:45, 60+:80$). We used these numbers to estimate 8$\times$8 age contact matrices from the online data on a daily basis. For a detailed schematic representation of the data collection pipeline see Fig. 3.

As we mentioned in the Discussion, it is likely that there are multiple types of biases present in the online data, such as recall bias, selection bias or social desirability bias. We do not expect that the weighting procedure can handle these biases (we applied it to improve the representativity of the data). However, it is important to emphasize that the proposed method of $R_t$ estimation works even if there are such biases present in the data, if biases are consistently present over time. As we scale with transmission rate and we focus on an epidemiologically homogeneous period, we can assume that these biases do not change over time, thus we believe that they do not significantly affect our estimation.

#### Representative data collection

As the participation in the online data collection was voluntary, respondents were not representative for the whole population of the country, moreover, their composition could change on a daily basis (see SI Figure 1). To account for these shortcomings and to record a representative sample, we started a smaller scale data collection campaign with different methodology but using the exact same questionnaire. This survey has been conducted with CATI (Computer Assisted Telephone Interview) survey technique by a public research company. The data collection started in April 2020 and has been repeated monthly. The respondents were selected by a multi-step stratified probability sampling technique from a database containing both mobile- and landline phone numbers. The sample is representative for the Hungarian adult (18 years old or older) population in terms of gender, age, education level and type of settlement; sampling errors were further corrected by post-stratification weights. Depending on the month, the numbers of recorded complete responses in each wave of the data collection were between 1, 000 and 1, 500, which fits the standard size of representative surveys in Hungary. The overall response rate was relatively high, $\sim 49\%$ as compared to other similar size surveys. In comparison, according to the data collection company, the average response rate of similar data collection methodologies at a nationally representative survey is between 15 and 20 percent. The collection of one wave generally took one week, where two-third of the responses corresponded to weekdays, and one-third to weekend days. Although telephone survey data has been collected once per month only with smaller sample size as compared to the online survey, it provided us with generalizable information about the contact patterns of the Hungarian adult population.

Taking the collected raw data we built up a data-cleaning pipeline to prepare the data for further analysis. This pipeline has been applied on both online and representative data. First, to avoid skewed averages due to outliers, we filtered survey answers if they contained very high out-of-home total proxy contact numbers added up for all age group. In this case we chose to drop the top 0.5 percentile of total contact numbers corresponding to a cut at more than 90 proxy contacts. Moreover, in the online survey we also omitted the answers from the analysis if the contact numbers have been larger than the average plus two standard deviations within the respondents’ own age group within the given time window or in the representative survey. In the exceptional cases when the number of responses within a time window for one age group was insufficient to calculate the standard deviation, we took an age-independent upper threshold computed from the system average. The latter filtering process was necessary for the online age-stratified matrices, since the $R_t$ calculation that was based on the spectral radius of the Next Generation Matrix method was very sensitive to sparse elements, and global filtering was unable to capture age group dependent outliers.

Because online responses for children did not contain information on their mask use, we estimated their contact numbers by re-scaling their reported contact numbers using their mask use percentages based on age and contact numbers from the representative survey of October 2020. Contact number is an important factor in this variable, because children tended to use masks in higher percentages in more crowded settings such as classrooms. Table 1 shows the mask use correction factors from the representative survey applied to the online responses of children.

**Table 1:** Mask use fractions of children based on age and contact number from representative survey.

Age	0–22 contacts	23+ contacts
3–6	0.1324	0.1509
7–10	0.2720	0.5635
11–14	0.3665	0.6979
15–17	0.3665	0.7100

#### Contact matrix reconstruction

To account for the non-representative biases in the online data, we worked out a method to dynamically estimate weights for each online respondent to re-weight the online data to create a close-to-representative population. For the selection of the weighting dimensions our goal was to identify socio-demographic variables with available population-level distributions, which were also present in our online questionnaire. First, we tested which variables affect the proxy contact numbers of the respondents using the representative survey. As the proxy contact numbers of the respondents can only be non-negative integers, we used negative binomial regression models on the first two waves (conducted in April and May, 2020) of the representative data collection together. Based on this model (see results in SI Table 1), we identified age, highest education level, region, type of settlement, and the interaction of gender and work status as significantly affecting the number of proxy contacts. For the validation of the weighting dimensions see Supplementary Information.

Using these variables and the latest census data^70, 71^, we calculated $w^x$ weights for every user *x* using the iterative proportional fitting method^72^ in each 7-day time window. This weighing methodology adjusts the cells of a contingency table created by the empirical distribution of the weighting dimensions in a way that their marginals fit to the expected distribution of the same dimensions. Empirical distributions were taken from the online survey, expected distributions were provided by the census data. One of the main advantages of this weighting methodology compared to standard cell weighting is to induce less likely extremely high or low weights - which could make the estimations unstable^73^. Thus, the actual weighted user sample within a one-week daily sliding window had marginals fitted to official census marginal distributions along the selected variables. We summarize this data construction pipeline in Fig. 3, while for a detailed description of a similar regression choice we refer to^23^.

Finally, to construct age-stratified contact matrices for each period, we categorized each respondent into eight age groups, namely $0-4$, $5-14$, $15-29$, $30-44$, $45-59$, $60-69$, $70-79$, and $80+$. We constructed $8\times 8$ matrices with column indices corresponding to the age group of the respondents and row indices correspond to the age group of their contacts. To formally define this matrix on the population level we follow the same procedure as described in^23^: Let assign by *X* be the set of respondents (ego), and by *Y* the set of individuals who are contacts of some $x\in X$. For a specific *x*, let $N_x \subset Y$ be the set of individuals who are contacts of *x*. We assign by $a(x)\in A=\{1,\dots ,8\}$ the age group of an individual *x*. We define the matrix $M^{x,y}$ for each $x\in X$ and $y \in N_x$ as $\left( M^{x,y}\right) _{i,j}=1$ if $a(x)=j$ and $a(y)=i$, and zero otherwise. For an ego *x* we can now compute its individual contact matrix as $\textrm{M}^x=\sum _{y\in N_x} M^{x,y}$. Finally, we use an individual weight $w^x$ assigned to each ego, coming from the IPF weighting method described above. This weight effectively describes how much an ego and its contacts should be considered in order to receive a contact matrix for a closer-to-representative population. Finally, the population level contact matrix is computed by ${{\textbf {M}}}=\sum _{x \in X} w^x \textrm{M}^x \big / \sum _{x \in X} w^x$.

### Ethical approval and consent to participate

We obtained fully informed consent from each participant before their enrolment in the study both in the case of online data collection and phone survey. In the online setting, anonymity of participants was ensured by using encrypted browser cookies to store hashed identifiers locally, while transferring only anonymous encrypted data to a central secure server. Encrypted browser cookies were used for the detection of returning respondents filling out the questionnaire on multiple days. The participants did not have to provide any information, which could be used for their re-identifcation. We did not involve participants under the age of 18 years in any of the data collections. Data collected about underaged groups were reported by their adult parents or legal guardians. The data collection was fully complying with the actual European and Hungarian privacy data regulations and was approved by the Hungarian National Authority for Data Protection and Freedom of Information, and also by the Health Science Council Scientific and Research Ethics Committee (resolution number IV/3073- 1 /2021/EKU). During our analysis all methods were performed in accordance with these relevant guidelines and regulations.

### Supplementary Information

Supplementary Information.

## Data Availability

In the repository https://github.com/zsvizi/r-eff-social-contact-surveys-covid-19-hungary^37^, we share all code and data necessary for the reproduction of our results. The shared data incorporates the source code for epidemic simulations and the data recording the empirical dynamical contact matrices. Other datasets are openly available as referenced in the text.

## References

[1] Van Bavel JJ, Baicker K, Boggio PS, Capraro V, Cichocka A, Cikara M (2020). Using social and behavioural science to support COVID-19 pandemic response. Nat. Hum. Behav..

[2] Betsch C (2020). How behavioural science data helps mitigate the COVID-19 crisis. Nat. Hum. Behav..

[3] Karsai, M., Koltai, J., Vásárhelyi, O., Röst, G. Hungary in Mask/MASZK in Hungary. *Corvinus J. Sociol. Soc. Policy*. 2 (2020).

[4] Perra, N. Non-pharmaceutical interventions during the COVID-19 pandemic: A review. Physics Reports. (2021). 10.1016/j.physrep.2021.02.001 PMC7881715 33612922

[5] Yıldırım M, Geçer E, Akgül Ö (2021). The impacts of vulnerability, perceived risk, and fear on preventive behaviours against COVID-19. Psychol. Health Med..

[6] Lim VW, Lim RL, Tan YR, Soh AS, Tan MX, Othman NB (2021). Government trust, perceptions of COVID-19 and behaviour change: Cohort surveys, Singapore. Bull. World Health Organ..

[7] Roozenbeek J, Schneider CR, Dryhurst S, Kerr J, Freeman AL, Recchia G (2020). Susceptibility to misinformation about COVID-19 around the world. Royal Soc. Open Sci..

[8] Kowalewski, M. Street protests in times of COVID-19: Adjusting tactics and marching ‘as usual’. Social Movement Studies. 1-8 (2020).

[9] Zhang J, Litvinova M, Liang Y, Wang Y, Wang W, Zhao S (2020). Changes in contact patterns shape the dynamics of the COVID-19 outbreak in China. Science.

[10] Elmer T, Mepham K, Stadtfeld C (2020). Students under lockdown: Comparisons of students’ social networks and mental health before and during the COVID-19 crisis in Switzerland. PLoS One.

[11] Warren, M.S., & Skillman, S.W. Mobility changes in response to COVID-19. arXiv preprint arXiv:2003.14228. (2020).

[12] Engle, S., Stromme, J., & Zhou, A. Staying at home: mobility effects of COVID-19. Available at SSRN 3565703. (2020).

[13] Leung K, Wu JT, Leung GM (2021). Real-time tracking and prediction of COVID-19 infection using digital proxies of population mobility and mixing. Nat. Commun..

[14] Naughton F, Ward E, Khondoker M, Belderson P, Marie Minihane A, Dainty J (2021). Health behaviour change during the UK COVID-19 lockdown: Findings from the first wave of the C-19 health behaviour and well-being daily tracker study. Br. J. Health. Psychol..

[15] Betsch, C., Wieler, L., Bosnjak, M., Ramharter, M., Stollorz, V., & Omer, S. et al. Germany COVID-19 Snapshot MOnitoring (COSMO Germany): Monitoring knowledge, risk perceptions, preventive behaviours, and public trust in the current coronavirus outbreak in Germany. PsychArchives. (2020).

[16] Kittel B, Kritzinger S, Boomgaarden H, Prainsack B, Eberl JM, Kalleitner F (2021). The Austrian Corona Panel Project: monitoring individual and societal dynamics amidst the COVID-19 crisis. Eur. Politic. Sci..

[17] Manica M, Guzzetta G, Riccardo F, Valenti A, Poletti P, Marziano V (2021). Impact of tiered restrictions on human activities and the epidemiology of the second wave of COVID-19 in Italy. Nat. Commun..

[18] Mossong J, Hens N, Jit M, Beutels P, Auranen K, Mikolajczyk R (2008). Social contacts and mixing patterns relevant to the spread of infectious diseases. PLoS Med..

[19] Wallinga J, Teunis P, Kretzschmar M (2006). Using data on social contacts to estimate age-specific transmission parameters for respiratory-spread infectious agents. Am. J. Epidemiol..

[20] Singh, R., & Adhikari, R. Age-structured impact of social distancing on the COVID-19 epidemic in India. arXiv preprint arXiv:2003.12055. (2020).

[21] Prem K, Cook AR, Jit M (2017). Projecting social contact matrices in 152 countries using contact surveys and demographic data. PLoS Comput. Biol..

[22] Mistry D, Litvinova M, Pastorey Piontti A, Chinazzi M, Fumanelli L, Gomes MF (2021). Inferring high-resolution human mixing patterns for disease modeling. Nat. Commun..

[23] Koltai J, Vásárhelyi O, Röst G, Karsai M (2021). Reconstructing social mixing patterns via weighted contact matrices from online and representative surveys. Sci. Rep..

[24] Hoang T, Coletti P, Melegaro A, Wallinga J, Grijalva CG, Edmunds JW (2019). A systematic review of social contact surveys to inform transmission models of close-contact infections. Epidemiology.

[25] Yc Fu (2007). Contact diaries: Building archives of actual and comprehensive personal networks. Field Methods.

[26] Munday JD, Jarvis CI, Gimma A, Wong KLM, van Zandvoort K (2021). Estimating the impact of reopening schools on the reproduction number of SARS-CoV-2 in England, using weekly contact survey data. BMC Med..

[27] Verelst F, Hermans L, Vercruysse S, Gimma A, Coletti P, Backer JA (2021). SOCRATES-CoMix: A platform for timely and open-source contact mixing data during and in between COVID-19 surges and interventions in over 20 European countries. BMC Med..

[28] CoMiX social contact data, http://www.socialcontactdata.org/data/ (date of access 2023.10.);.

[29] Munday JD, Abbott S, Meakin S, Funk S (2023). Evaluating the use of social contact data to produce age-specific short-term forecasts of SARS-CoV-2 incidence in England. PLoS Comput. Biol..

[30] Keeling MJ, Hollingsworth TD, Read JM (2020). Efficacy of contact tracing for the containment of the 2019 novel coronavirus (COVID-19). J. Epidemiol. Commun. Health.

[31] Delamater PL, Street EJ, Leslie TF, Yang YT, Jacobsen KH (2019). Complexity of the basic reproduction number ($$R_0$$). Emerg. Infect. Dis..

[32] Dietz K (1993). The estimation of the basic reproduction number for infectious diseases. Stat. Methods Med. Res..

[33] Cori A, Ferguson NM, Fraser C, Cauchemez S (2013). A New framework and software to estimate time-varying reproduction numbers during epidemics. Am. J. Epidemiol..

[34] Wallinga J, Lipsitch M (2007). How generation intervals shape the relationship between growth rates and reproductive numbers. Proc. Royal Soc. B: Biol. Sci..

[35] Organization, W.H., et al. Public health criteria to adjust public health and social measures in the context of COVID-19: annex to considerations in adjusting public health and social measures in the context of COVID-19, 12 May 2020. World Health Organization; (2020).

[36] Hasell J, Mathieu E, Beltekian D, Macdonald B, Giattino C, Ortiz-Ospina E (2020). A cross-country database of COVID-19 testing. Sci. Data.

[37] Code and data repository for estimated daily age-contact matrices, https://github.com/zsvizi/r-eff-social-contact-surveys-covid-19-hungary;.

[38] MASZK - Hungarian Data Provider Questionnaire, https://figshare.com/articles/online_resource/Hungarian_Data_Provider_Questionnaire/13550057;.

[39] Hale T, Angrist N, Goldszmidt R, Kira B, Petherick A, Phillips T (2021). A global panel database of pandemic policies (Oxford COVID-19 Government Response Tracker). Nat. Hum. Behav..

[40] Oroszi B, Horváth JK, Túri G, Krisztalovics K, Röst G (2021). Az epidemiológiai surveillance és járványmatematikai előrejelzések szerepe a pandémiás hullámok megelőzésében, mérséklésében-hol tartunk most, és hová kellene eljutni. Scientia et Securitas..

[41] Diekmann O, Heesterbeek JAP, Metz JA (1990). On the definition and the computation of the basic reproduction ratio $$R_0$$ in models for infectious diseases in heterogeneous populations. J. Math. Biol..

[42] Röst G, Bartha FA, Bogya N, Boldog P, Dénes A, Ferenci T (2020). Early phase of the COVID-19 outbreak in Hungary and post-lockdown scenarios. Viruses.

[43] Ferenci, T. The real-time epidemiology of the Hungarian coronavirus pandemic https://research.physcon.uni-obuda.hu/COVID19MagyarEpi/ (date of access 2022.07.07);.

[44] Sam, A., Joel, H., Katharine, S., Katelyn, G., Joe, H., Hamada, S. B., et al. EpiNow2: Estimate real-time case counts and time-varying epidemiological parameters, (2020).

[45] Parag KV (2021). Improved estimation of time-varying reproduction numbers at low case incidence and between epidemic waves. PLoS Comput. Biol..

[46] Gressani O, Wallinga J, Althaus CL, Hens N, Faes C (2022). EpiLPS: A fast and flexible Bayesian tool for estimation of the time-varying reproduction number. PLoS Comput. Biol..

[47] Kostkova P, Saigí-Rubió F, Eguia H, Borbolla D, Verschuuren M, Hamilton C (2021). Data and digital solutions to support surveillance strategies in the context of the COVID-19 pandemic. Front. Digital Health.

[48] Jarvis CI, Van Zandvoort K, Gimma A, Prem K, Klepac P (2020). Quantifying the impact of physical distance measures on the transmission of COVID-19 in the UK. BMC Med..

[49] Dugas AF, Jalalpour M, Gel Y, Levin S, Torcaso F, Igusa T (2013). Influenza forecasting with Google flu trends. PLoS One.

[50] Tang L, Bie B, Park SE, Zhi D (2018). Social media and outbreaks of emerging infectious diseases: A systematic review of literature. Am. J. Infect. Control.

[51] Perrotta, D., Tizzoni, M., & Paolotti, D. Using participatory Web-based surveillance data to improve seasonal influenza forecasting in Italy. In: Proceedings of the 26th International Conference on World Wide Web; (2017). p. 303-10.

[52] Perrotta D, Bella A, Rizzo C, Paolotti D (2017). Participatory online surveillance as a supplementary tool to sentinel doctors for influenza-like illness surveillance in Italy. PLoS One.

[53] Koppeschaar CE, Colizza V, Guerrisi C, Turbelin C, Duggan J, Edmunds WJ (2017). Influenzanet: Citizens among 10 countries collaborating to monitor influenza in Europe. JMIR Public Health Surveill..

[54] Pullano G, Di Domenico L, Sabbatini CE, Valdano E, Turbelin C, Debin M (2021). Underdetection of cases of COVID-19 in France threatens epidemic control. Nature.

[55] Kjelsø C, Galle M, Bang H, Ethelberg S, Krause TG (2016). Influmeter-an online tool for self-reporting of influenza-like illness in Denmark. Infect. Dis..

[56] Brownstein JS, Chu S, Marathe A, Marathe MV, Nguyen AT, Paolotti D (2017). Combining participatory influenza surveillance with modeling and forecasting: Three alternative approaches. JMIR Public Health Surveill..

[57] Lazer D, Kennedy R, King G, Vespignani A (2014). The parable of Google Flu: Traps in big data analysis. Science.

[58] Vanni F, Lambert D, Palatella L, Grigolini P (2021). On the use of aggregated human mobility data to estimate the reproduction number. Sci. Rep..

[59] Jung SM, Endo A, Akhmetzhanov AR, Nishiura H (2021). Predicting the effective reproduction number of COVID-19: Inference using human mobility, temperature, and risk awareness. Int. J. Infectious Dis..

[60] Gozzi N (2022). Anatomy of the first six months of COVID-19 Vaccination campaign in Italy. PLOS Comput. Biol..

[61] Bao R, Zhang A (2020). Does lockdown reduce air pollution? Evidence from 44 cities in Northern China. Sci. Total Environ..

[62] Szocska M, Pollner P, Schiszler I, Joo T, Palicz T, McKee M (2021). Countrywide population movement monitoring using mobile devices generated (big) data during the COVID-19 crisis. Sci. Rep..

[63] Wang S, Liu Y, Hu T (2020). Examining the change of human mobility adherent to social restriction policies and its effect on COVID-19 cases in Australia. Int. J. Environ. Res. Public Health.

[64] Gottumukkala R, Katragadda S, Bhupatiraju RT, Kamal AM, Raghavan V, Chu H (2021). Exploring the relationship between mobility and COVID- 19 infection rates for the second peak in the United States using phase-wise association. BMC Public Health.

[65] Bokányi E, Pollner P, Joó T (2021). Kontaktkutatás, vezetői információs rendszer. Scientia et Securitas.

[66] Larsen, M., Nyrup, J., & Petersen, M.B. et al. Do survey estimates of the public’s compliance with COVID-19 regulations suffer from social desirability bias? *J. Behavioral Public Adm.* **3**(2) (2020).

[67] Jensen, U.T. et al. Is self-reported social distancing susceptible to social desirability bias? Using the crosswise model to elicit sensitive behaviors. *J. Behavioral Public Adm.* **3**(2) (2020).

[68] Hungarian Data Supply Questionnaire (MASZK) Team, https://covid.sed.hu/tabs/staff, (date of access 2022.07.03);.

[69] Nemzeti Adatvédelmi és Információszabadság Hatóság, https://www.naih.hu (date of access 2020.12.);.

[70] Office HCS. Hungarian Census 2011, http://www.ksh.hu/nepszamlalas/ (date of access 2020.09.28);.

[71] Office HCS. Hungarian Microensus 2016, https://www.ksh.hu/mikrocenzus2016/(date of access 2020.09.28);.

[72] Bishop YM, Fienberg SE, Holland PW (2007). Discrete Multivariate Analysis: Theory and Practice.

[73] Lavrakas PJ (2008). Encyclopedia of Survey Research Methods.

