# Supplementary material for: Real-time estimation of the effective reproduction number of COVID-19 from behavioral data
Source: Sci Rep. 2023 Dec 5;13:21452. doi: 10.1038/s41598-023-46418-z (PMC10698193; doi:10.1038/s41598-023-46418-z)
Supplement: Supplementary file 1 — Supplementary Information. [file 41598_2023_46418_MOESM1_ESM.pdf]

Supplementary Materials

Real-time estimation of the effective reproduction number of COVID-19 from behavioral data

E. Bokányi, Zs. Vizi, J. Koltai, G. Röst, and M. Karsai

Validation of the weighting dimensions

To identify the main weighting dimensions for the iterative proportional fitting, Table 1 shows the results of the negative binomial regression model on the proxy contact numbers of the respondents. Here the independent variables are the dimensions with available population-level distribution, and which were also present in the questionnaire. We can observe that in each dimension, there is at least one category that significantly affects the proxy contact numbers compared to the reference category. These results suggest that all dimensions from this regression model should be considered as weighting dimensions on the online data since they all influence the contact patterns of people.

**Independent Variables**	**B**	**Std. Error**	**Wald Chi-Square**	**Significance**
Intercept	0.270	0.098	7.61	0.006
19-29 years old	0.846	0.072	137.24	0.000
30-44 years old	0.983	0.063	242.37	0.000
45-59 years old	0.964	0.062	240.67	0.000
ref: 60 years old or older				
max. vocation	0.078	0.062	1.58	0.209
secondary education	0.212	0.058	13.17	0.000
ref: higher education				
other region	0.143	0.066	4.72	0.030
ref: Central Hungary				
Budapest	-0.096	0.083	1.34	0.248
county town	-0.167	0.064	6.74	0.009
city	-0.128	0.052	6.16	0.013
ref: village				
man works	0.756	0.055	189.55	0.000
man does not work	0.386	0.066	33.99	0.000
woman works	0.661	0.063	111.57	0.000
ref: woman does not work				

Table 1: Negative binomial regression model on the number of contacts with the potential weighting dimensions as independent variables

Change in the population composition

In the online survey, users were not representative of the population of Hungary. Moreover, their composition could change on a daily basis. Figure 1 shows the composition as a function of time throughout the time period of this analysis alongside with the representative percentages obtained from official statistics of the Central Bureau of Statistics of Hungary (KSH) [4, 5]. Variables correspond to the significant

dimensions that have been determined from the representative survey regressions, and that have the largest influence on people's contact behavior.

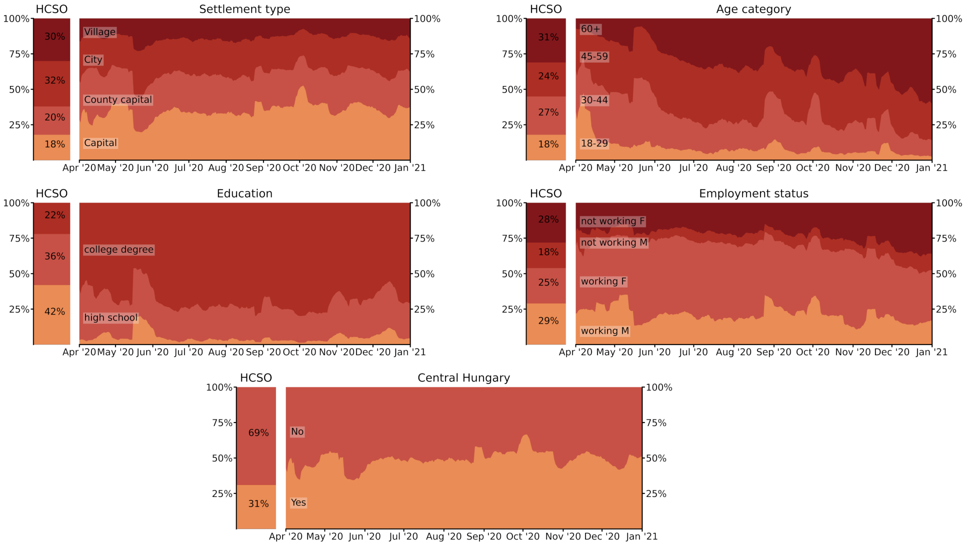

Figure 1: Distributions of users with respect to the demographic attributes used for the IPF weighting in the online survey. Reference percentages representative of the adult population obtained from official census data [4, 5] are to the left of each subplot labeled as HCSO (Hungarian Central Statistical Office). Central Hungary refers to whether a user lives in the EU region of the capital city, Budapest, and its surrounding county, called "Pest megye". Employment status is measured for both genders, M=male and F=female.

Epidemic model

For investigating the dynamics of the COVID-19 epidemics, we use a slightly modified deterministic model from [6]. In the following, we briefly introduce the mechanisms of the epidemic model. This model is defined on a population of people where we denote by S the susceptibles, i.e. who can contract the disease. If individuals who get contracted the disease first get latent (L), i.e. carry the virus, but they have no symptoms yet. Then a large fraction of the latents transit to the class for asymptomatic cases, i.e. having at most mild symptoms (denoted by M), but with the ability to infecting susceptible individuals. Others develop more severe symptoms, they proceed first to the pre-symptomatic (P), then to the infected compartment (I). Individuals from A compartment will all recover and consequently proceed to class for recovered (denoted by R), while symptomatically infected individuals may either recover without requiring further treatment or become hospitalized.

As we have seen in the COVID-19 pandemic, it is of high importance to be able to estimate number of hospital beds and intensive care unit (ICU) beds, thus we differentiate symptomatically infected individuals who need hospital and critical care (ICU), denoted by H and C, respectively. We assume that patients admitted to non-intensive treatment will all recover, thus proceed to class R, however, fatal outcome may occur for individuals from class I_c implying the transition from C to the D compartment. Those who are out of ICU and on the path to recovery are first collected in the compartment C_r from where they proceed to class R.

Additionally, we assume that the latency and infectious periods are gamma distributed, and for their modeling we use so called the linear chain trick, i.e. we divide classes L, A and I into further compartments. For gamma distributed latency period with Erlang parameter $m = 2$, we introduce classes L_1 and L_2, for infectious period with Erlang parameter $m = 3$, for asymptomatically and symptomatically infected individuals we have M_1, M_2, M_3 and I_1, I_2, I_3 compartments, respectively.

Considering all the introduced compartments, the dynamics of the system is described by the following

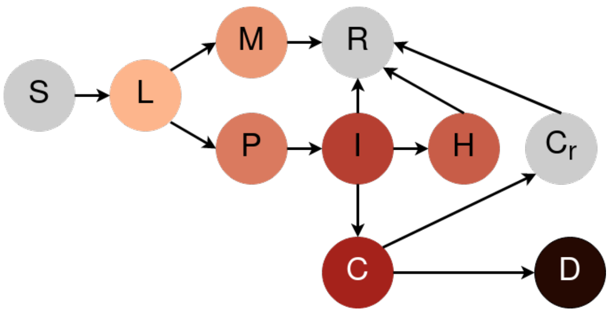

Figure 2: Compartment transmission diagram of the epidemic model

differential equation system:

$$
\begin{aligned}
S^{a\prime}(t) &= -\frac{S^a(t)}{N^a(t)}\sigma^a \sum_{k\in\{1,\dots,8\}} \left[\beta_{\mathbf{p}}^{(k,i)} I_{\mathbf{p}}^k(t) + \sum_{\mathbf{j}\in\{\mathbf{m},\mathbf{i}\}\times\{1,2,3\}} \beta_{\mathbf{j}}^{(k,i)} I_{\mathbf{j}}^k(t) \right], \\
L_{\mathbf{1}}^{a\prime}(t) &= \frac{S^a(t)}{N^a(t)}\sigma^a \sum_{k\in\{1,\dots,8\}} \left[\beta_{\mathbf{p}}^{(k,i)} I_{\mathbf{p}}^k(t) + \sum_{\mathbf{j}\in\{\mathbf{m},\mathbf{i}\}\times\{1,2,3\}} \beta_{\mathbf{j}}^{(k,i)} I_{\mathbf{j}}^k(t) \right] - \alpha_{\mathbf{l},\mathbf{1}}^a L_{\mathbf{1}}^a(t), \\
L_{\mathbf{2}}^{a\prime}(t) &= \alpha_{\mathbf{l},\mathbf{1}}^a L_{\mathbf{1}}^a(t) - \alpha_{\mathbf{l},\mathbf{2}}^a L_{\mathbf{2}}^a(t), \\
I_{\mathbf{m},\mathbf{1}}^{a\prime}(t) &= p^a \alpha_{\mathbf{l},\mathbf{2}}^a L_{\mathbf{2}}^a(t) - \gamma_{\mathbf{m},\mathbf{1}}^a I_{\mathbf{m},\mathbf{1}}^a(t), \\
I_{\mathbf{m},\mathbf{2}}^{a\prime}(t) &= \gamma_{\mathbf{m},\mathbf{1}}^a I_{\mathbf{m},\mathbf{1}}^a(t) - \gamma_{\mathbf{m},\mathbf{2}}^a I_{\mathbf{m},\mathbf{2}}^a(t), \\
I_{\mathbf{m},\mathbf{3}}^{a\prime}(t) &= \gamma_{\mathbf{m},\mathbf{2}}^a I_{\mathbf{m},\mathbf{2}}^a(t) - \gamma_{\mathbf{m},\mathbf{3}}^a I_{\mathbf{m},\mathbf{3}}^a(t), \\
I_{\mathbf{p}}^{a\prime}(t) &= (1-p^a)\alpha_{\mathbf{l},\mathbf{2}}^a L_{\mathbf{2}}^a(t) - \alpha_{\mathbf{p}}^a I_{\mathbf{p}}^a(t), \\
I_{\mathbf{i},\mathbf{1}}^{a\prime}(t) &= \alpha_{\mathbf{p}}^a I_{\mathbf{p}}^a(t) - \gamma_{\mathbf{i},\mathbf{1}}^a I_{\mathbf{i},\mathbf{1}}^a(t), \\
I_{\mathbf{i},\mathbf{2}}^{a\prime}(t) &= \gamma_{\mathbf{i},\mathbf{1}}^a I_{\mathbf{i},\mathbf{1}}^a(t) - \gamma_{\mathbf{i},\mathbf{2}}^a I_{\mathbf{i},\mathbf{2}}^a(t), \\
I_{\mathbf{i},\mathbf{3}}^{a}(t) &= \gamma_{\mathbf{i},\mathbf{2}}^a I_{\mathbf{i},\mathbf{2}}^a(t) - \gamma_{\mathbf{i},\mathbf{3}}^a I_{\mathbf{i},\mathbf{3}}^a(t), \\
I_{\mathbf{h}}^{a\prime}(t) &= h^a(1-\xi^a)\gamma_{\mathbf{i},\mathbf{3}}^a I_{\mathbf{i},\mathbf{3}}^a(t) - \gamma_{\mathbf{h}}^a I_{\mathbf{h}}^a(t), \\
I_{\mathbf{c}}^{a\prime}(t) &= h^a\xi^a\gamma_{\mathbf{i},\mathbf{3}}^a I_{\mathbf{i},\mathbf{3}}^a(t) - \gamma_{\mathbf{c}}^a I_{\mathbf{c}}^a(t), \\
I_{\mathbf{cr}}^{a\prime}(t) &= (1-\mu^a)\gamma_{\mathbf{c}}^a I_{\mathbf{c}}^a(t) - \gamma_{\mathbf{cr}}^a I_{\mathbf{cr}}^a(t), \\
R^{a\prime}(t) &= \gamma_{\mathbf{m},\mathbf{3}}^a I_{\mathbf{m},\mathbf{3}}^a(t) + (1-h^a)\gamma_{\mathbf{i},\mathbf{3}}^a I_{\mathbf{i},\mathbf{3}}^a(t) + \gamma_{\mathbf{h}}^a I_{\mathbf{h}}^a(t) + \gamma_{\mathbf{cr}}^a I_{\mathbf{cr}}^a(t), \\
D^{a\prime}(t) &= \mu^a\gamma_{\mathbf{c}}^a I_{\mathbf{c}}^a(t),
\end{aligned} \tag{1}
$$

where the index $a \in \{1,\dots,8\}$ represents the corresponding age group.

Since we want to take into account the different characteristics of the disease in various age groups, we stratified the Hungarian population into eight groups, using the same age structure that we used in the questionnaires. The model parameters are calibrated based on comprehensive literature review and they are aligned to private data provided by the National Public Health Center in Hungary (for a previously published parameter set, see [6]). Since the previously mentioned model was parameterized for seven age groups, we slightly changed the parameter vectors as shown in Table 2. Actually, we added another aspect to the disease transmission term, which considers age-dependent susceptibility σ of individuals: we set this value to 1.0 except for the first two age groups, for which we use 0.5. This aligns with the observations that children are less likely to get infected at the contact with an infected individual.

Simulating an epidemic model requires determining the epidemiological parameters along with initial state at the start of the simulation. In our analysis, we assume that for the first wave we do not have population-level epidemic spread, however, for the second wave we consider that 1% of the population was infected during the first wave, and an additional 1% was recovered from an outbreak started already in the summer, i.e. we already have infected individuals distributed over all age groups. The latter approach enables us to pass proper initial values for the deterministic model.

Probability / Age group		0–4	5–14	15–29	30–44	45–59	60–69	70–79	80–
Asymptomatic course	p^a	0.9	0.8	0.7	0.6	0.4	0.3	0.2	0.1
Hospitalization or intensive care (from I_3^a)	h^a	0.00045	0.00045	0.0041	0.0028	0.1094	0.2529	0.4663	0.4965
Intensive care (given hospitalization)	ξ^a	0.333	0.333	0.312	0.297	0.292	0.293	0.293	0.293
Fatal outcome (from C_r^a)	μ^a	0.2	0.2	0.216	0.25	0.582	0.678	0.687	0.7
Susceptibility parameter	σ^a	0.5	0.5	1.0	1.0	1.0	1.0	1.0	1.0

Table 2: Age-dependent epidemiological parameters of COVID-19 for eight age groups

Effective reproduction number

The transmission part of an epidemic model depends on the contact patterns between people in the susceptible and infected compartments, and the probability of virus transmission during the contacts, which can differ for different infectious compartments. This implies that we have to give estimates for $\beta_X^{(k,a)}$, which corresponds to the transmission rate of an infectious individual from X and age group k at contact with a susceptible from age group a, where $X \in \{P, M_1, M_2, M_3, I_1, I_2, I_3\}$. For this purpose, we compute the Next Generation Matrix (NGM) and baseline transmission rate β_0 using techniques of [2]. Since the actually defined model only slightly differs from the one in [6], we might omit the detailed elaboration and highlight only the components have to be changed for the calculations, whereas the scheme of the computation remains the same.

For now, the block-diagonal transitional matrix Σ has to be modified at elements $(3,2), (4,2), (4,3)$ and $(7,3)$, therefore we have

$$\Sigma_a = \begin{bmatrix} -\alpha_{\mathbf{l,1}}^a & 0 & 0 & 0 & 0 & 0 & 0 & 0 & 0 \\ \alpha_{\mathbf{l,1}}^a & -\alpha_{\mathbf{l,2}}^a & 0 & 0 & 0 & 0 & 0 & 0 & 0 \\ 0 & (1-p^a)\alpha_{\mathbf{l,2}}^a & -\alpha_{\mathbf{p}}^a & 0 & 0 & 0 & 0 & 0 & 0 \\ 0 & p^a\alpha_{\mathbf{l,2}}^a & 0 & -\gamma_{\mathbf{m,1}}^a & 0 & 0 & 0 & 0 & 0 \\ 0 & 0 & 0 & \gamma_{\mathbf{m,1}}^a & -\gamma_{\mathbf{m,2}}^a & 0 & 0 & 0 & 0 \\ 0 & 0 & 0 & 0 & \gamma_{\mathbf{m,2}}^a & -\gamma_{\mathbf{m,3}}^a & 0 & 0 & 0 \\ 0 & 0 & \alpha_{\mathbf{p}}^a & 0 & 0 & 0 & -\gamma_{\mathbf{i,1}}^a & 0 & 0 \\ 0 & 0 & 0 & 0 & 0 & 0 & \gamma_{\mathbf{i,1}}^a & -\gamma_{\mathbf{i,2}}^a & 0 \\ 0 & 0 & 0 & 0 & 0 & 0 & 0 & \gamma_{\mathbf{i,2}}^a & -\gamma_{\mathbf{i,3}}^a \end{bmatrix}$$

for $a = 1, \ldots, 8$.

The transmission matrix $\mathbf{T}$ is affected by the above mentioned age-dependent susceptibility parameter for the first two age groups (since these parameters differ from 1). In these cases, we have to multiply all elements of the building blocks $\mathbf{T}_1$ and $\mathbf{T}_2$ with the respective susceptibility parameter value (that we assumed 0.5 for both age groups, see discussion above).

Using NGM methodology, for a given initial contact matrix and observed R_t, we are able to estimate the baseline transmission rate finalizing the parametrization of the epidemic model. For calculating the effective reproduction number, on the one hand, we update the online measured contact matrix on a daily basis. On the other hand, for the second wave (when the virus spread across the whole country), we have to scale the elements of the contact matrices by the proportion of the susceptibles actually given by the model. We perform this as multiplying each column of the matrix by the respective proportion value.

Seasonality effects

Since we investigate a nine-month period of 2020, we cannot neglect the effect of seasonal changes, which is incorporated into the model via scaling the baseline transmission rate by a time-varying function called seasonality function. This function is usually chosen for a 1-year periodic sine or cosine function [6, 1], but our experimental observations from modeling the pandemic in Hungary show that the function shown in Figure 3 aligns better with the epidemic data. Clearly, during warmer periods of the year (from end

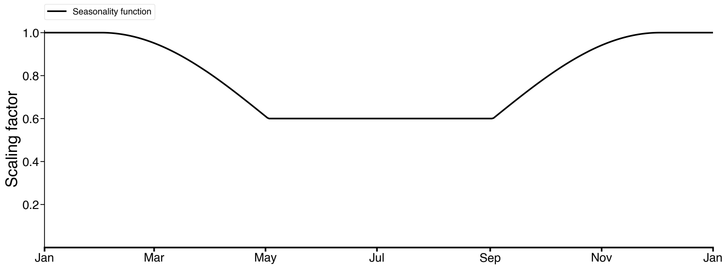

Figure 3: Seasonality function

of the spring until early autumn) the transmission rate is reduced in the population e.g. due to weather conditions and better natural ventilation, while a ramp-up and ramp-down phase are considered after and before this period, respectively. Furthermore during the winter and summer periods the efficiency rate does not change significantly, thus we assume that this rate is constant over these time intervals. Finally we set the ratio of 0.6 between summer and winter time [1, 3] and we kept the sinusoidal change in the complementary part for a year.

References

[1] Duygu Balcan, Hao Hu, Bruno Goncalves, Paolo Bajardi, Chiara Poletto, Jose J Ramasco, Daniela Paolotti, Nicola Perra, Michele Tizzoni, Wouter Van den Broeck, et al. Seasonal transmission potential and activity peaks of the new influenza A (H1N1): a Monte Carlo likelihood analysis based on human mobility. *BMC Medicine*, 7(1):1–12, 2009.

[2] Odo Diekmann, Johan Andre Peter Heesterbeek, and Johan AJ Metz. On the definition and the computation of the basic reproduction ratio r_0 in models for infectious diseases in heterogeneous populations. *Journal of Mathematical Biology*, 28(4):365–382, 1990.

[3] Nicolò Gozzi, Matteo Chinazzi, Jessica T Davis, Kunpeng Mu, Ana Pastore y Piontti, Marco Ajelli, Nicola Perra, and Alessandro Vespignani. Anatomy of the first six months of COVID-19 vaccination campaign in Italy. *PLOS Computational Biology*, 18(5):e1010146, 2022.

[4] Hungarian Central Statistical Office. Hungarian census 2011, http://www.ksh.hu/nepszamlalas/ (date of access 2020.09.28).

[5] Hungarian Central Statistical Office. Hungarian microensus 2016, https://www.ksh.hu/mikrocenzus2016/ (date of access 2020.09.28).

[6] Gergely Röst, Ferenc A. Bartha, Norbert Bogya, Péter Boldog, Attila Dénes, Tamás Ferenci, Krisztina J. Horváth, Attila Juhász, Csilla Nagy, Tamás Tekeli, Zsolt Vizi, and Beatrix Oroszi. Early phase of the COVID-19 outbreak in Hungary and post-lockdown scenarios. *Viruses*, 12(7), 2020.
